# DNA/RNA heteroduplex oligonucleotide for highly efficient gene silencing

**DOI:** 10.1038/ncomms8969

**Published:** 2015-08-10

**Authors:** Kazutaka Nishina, Wenying Piao, Kie Yoshida-Tanaka, Yumiko Sujino, Tomoko Nishina, Tsuyoshi Yamamoto, Keiko Nitta, Kotaro Yoshioka, Hiroya Kuwahara, Hidenori Yasuhara, Takeshi Baba, Fumiko Ono, Kanjiro Miyata, Koichi Miyake, Punit P. Seth, Audrey Low, Masayuki Yoshida, C. Frank Bennett, Kazunori Kataoka, Hidehiro Mizusawa, Satoshi Obika, Takanori Yokota

**Affiliations:** 1 Department of Neurology and Neurological Science, Graduate School, Tokyo Medical and Dental University, 1-5-45 Yushima, Bunkyo-ku, Tokyo 113-8519, Japan; 2 Section of Molecular Technology, Core Research for Evolutional Science and Technology (CREST), Japan Science and Technology Agency (JST), 4-1-8 Honcho, Kawaguchi-shi, Saitama 332-0012, Japan; 3 Bioorganic Chemistry, Graduate School of Pharmaceutical Sciences, Osaka University, 1-6 Yamadaoka, Suita-shi, Osaka 565-0871, Japan; 4 The Corporation for Production and Research of Laboratory Primates, 1-16-2 Sakura, Tsukuba-shi, Ibaraki 305-0003, Japan; 5 Division of Clinical Biotechnology, Center for Disease Biology and Integrative Medicine, Graduate School of Medicine, The University of Tokyo, 7-3-1 Hongo, Bunkyo-ku, Tokyo 113-0033, Japan; 6 Department of Biochemistry and Molecular Biology, Nippon Medical School, 1-1-5 Sendagi, Bunkyo-ku, Tokyo 113-8602, Japan; 7 Isis Pharmaceuticals, 2855 Gazelle Court, Carlsbad, California 92010, USA; 8 Department of Life Science and Medical Ethics, Graduate School, Tokyo Medical and Dental University, 1-5-45 Yushima, Bunkyo-ku, Tokyo 113-8519, Japan; 9 Department of Materials Engineering, Graduate School of Engineering, The University of Tokyo, 7-3-1 Hongo, Bunkyo-ku, Tokyo 113-8656, Japan

## Abstract

Antisense oligonucleotides (ASOs) are recognized therapeutic agents for the modulation of specific genes at the post-transcriptional level. Similar to any medical drugs, there are opportunities to improve their efficacy and safety. Here we develop a short DNA/RNA heteroduplex oligonucleotide (HDO) with a structure different from double-stranded RNA used for short interfering RNA and single-stranded DNA used for ASO. A DNA/locked nucleotide acid gapmer duplex with an α-tocopherol-conjugated complementary RNA (Toc-HDO) is significantly more potent at reducing the expression of the targeted mRNA in liver compared with the parent single-stranded gapmer ASO. Toc-HDO also improves the phenotype in disease models more effectively. In addition, the high potency of Toc-HDO results in a reduction of liver dysfunction observed in the parent ASO at a similar silencing effect. HDO technology offers a novel concept of therapeutic oligonucleotides, and the development of this molecular design opens a new therapeutic field.

## Abstract (web summary)

Antisense oligonucleotides (ASOs) can repress the expression of specific genes. Here, the authors show that a DNA/RNA heteroduplex oligonucleotide (HDO) with a structure different from ASOs is more potent in suppressing target gene expression, and causes a less adverse effect in mouse liver.

## Graphical abstract

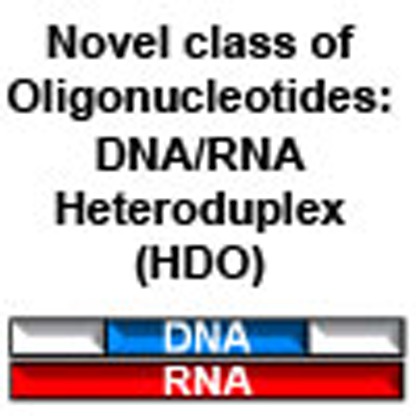

Two major types of oligonucleotide drugs are currently being developed as therapeutic platforms for the reduction of target gene expression: short interfering RNA (siRNA) and RNase H-dependent single-stranded antisense oligonucleotides (ASOs)^1^,^2^,^3^,^4^,^5^,^6^,^7^. The use of RNA interference is a promising therapeutic strategy, but the double-stranded RNAs appear to be less efficient in distribution to tissues and in cellular uptake compared with single-stranded ASOs. To address this issue, siRNAs are often formulated into polymers, lipid nanoparticles (LNPs) or conjugated to targeting ligands including lipid or ligands of cell surface receptors^8^,^9^,^10^,^11^,^12^. Several formulated siRNAs are now in clinical trials, which will define the safety and efficacy of these agents^12^,^13^. Single-stranded ASOs have demonstrated distribution to tissues and cellular uptake in the absence of formulations or targeting ligands. There are multiple mechanisms by which single-stranded ASOs can be used to affect the processing and metabolism of the target RNA, including degradation of the target RNA via the recruitment of endogenous ribonuclease (RNase) H1 (refs 14, 15). Chemical modifications, particularly the use of locked nucleic acid (LNA)^16^,^17^,^18^, 2′-*O*-methoxyethyl RNA (2′-MOE)^19^,^20^ and 2′,4′-constrained 2′-*O*-ethyl bridged nucleic acid (cEt)^21^,^22^ on the ends of the ASO markedly improve ASO binding affinity for the target messenger RNA (mRNA), resulting in increased activity. Kynamro (Mipomersen), a second-generation ASO-targeting Apolipoprotein B (*ApoB*) mRNA, was recently approved by the US Food and Drug Administration for the treatment of familial hypercholesterolaemia^6^,^7^. In addition, high-affinity chemical modifications enable the design of short ASOs that possess good potency compared with longer oligonucleotides^23^.

Despite progress in the design of new chemical modifications of oligonucleotides, methods that further improve the potency of oligonucleotide drugs and improve safety and tolerability are highly desirable. The insufficient delivery, poor cellular uptake of ASOs and their inefficient access to target RNA are major impediments to *in vivo* silencing^24^. Previously, we conjugated α-tocopherol, a natural isomer of vitamin E, to siRNA and obtained increased delivery of siRNA in mouse liver and brain^25^,^26^. We tried to introduce α-tocopherol directly into single-stranded ASOs as a delivery system for ASOs. However, we found that the chemical method for conjugation to oligonucleotides was critical, as lipid needed to be released from the oligonucleotide to allow the oligonucleotide to traffic to the target mRNA^27^.

Here we describe the novel observation that heteroduplex oligonucleotide (HDO) consisting of a DNA/LNA gapmer and its complementary RNA (cRNA) conjugated to a lipid enhances the activity of the gapmer ASO. Lipids, including α-tocopherol, can be covalently bound to the RNA strand of HDO. The RNA strand is cleaved in cells and tissues by a nuclease releasing the single-stranded ASO in the target cells, allowing it to bind to the targeted RNA. We demonstrate enhanced potency and better tolerability of α-tocopherol-conjugated HDO (Toc-HDO) in comparison with parent single-stranded ASO.

## Results

### Structure of the Toc-HDO

**Figure 1:**
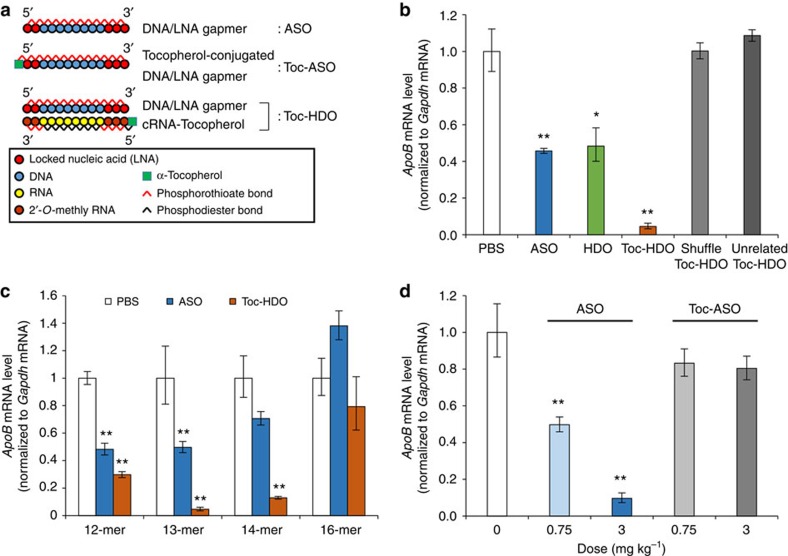
Design and effects of Toc-HDO. (**a**) Schematic illustration of the construction of ASO, Toc-ASO and Toc-HDO. (**b**,**c**) The effects of Toc-HDO on *ApoB* mRNA levels in mouse liver 3 d after injection by qRT–PCR analyses. (**b**) After injection of 0.75 mg kg^−1^ ASO, HDO or Toc-HDO-targeting *ApoB*, shuffle sequence of Toc-HDO-targeting *ApoB* or Toc-HDO targeting an unrelated gene (*n*=3, mean values±s.e.m., *P<0.05, **P<0.01 versus PBS). (**c**) After injection of a different length of 0.75 mg kg^−1^ ASO or Toc-HDO-targeting *ApoB* (*n*=3, mean values±s.e.m., **P<0.01 versus PBS). (**d**) The effects of Toc-ASO on *ApoB* mRNA levels in mouse liver 3 d after injection of 0.75 or 3 mg kg^−1^ ASO- or Toc-ASO-targeting *ApoB* by qRT–PCR analyses (*n*=3, mean values±s.e.m., **P<0.01 versus PBS). Data are representative of at least three independent experiments each (**b**–**d**). *P* values were calculated from the Student's two-tailed *t*-test (**b**–**d**).

We designed a Toc-HDO (Fig. 1a). The DNA strand is composed of gapmer ASO in which 8–10 DNA oligonucleotides are flanked by 2–3 LNA oligonucleotides, and all internucleotide linkages were modified by phosphorothioate substitution. The RNA strand is complementary to the DNA strand (cRNA), and it is bound to α-tocopherol on the 5′-end. In the RNA strand, phosphorothioate-modified 2′-*O*-methyl RNA is used for the nucleotides complementary to LNA in the DNA strand for protection from exonucleases, whereas the nucleotides in the centre portion complementary to the DNA are unmodified natural RNAs.

### Silencing efficacy of the Toc-HDO in mice

When Toc-HDO-targeting endogenous mouse *ApoB* mRNA (0.75 mg kg^−1^) was intravenously injected into mice, it produced a greater reduction of *ApoB* mRNA (95%) compared with an equivalent dose of the parent ASO without α-tocopherol (Fig. 1b). A similar finding was observed using a different ASO-targeting *ApoB* mRNA (Supplementary Fig. 1). The Toc-HDO in which the sequence of the ASO was scrambled or the Toc-HDO that targeted an unrelated gene did not reduce *ApoB* mRNA levels (Fig. 1b). The effect of ASO length was studied by varying the length of the ASO from 12 to 16 nucleotides (melting temperatures are shown in Supplementary Table 1). As previously reported^23^, 12- and 13-mer LNA-modified ASOs were effective at reducing *ApoB* mRNA, with activity decreasing with 14- and 16-mers (Fig. 1c). The most effective length for the duplexed HDO was 13 nucleotides, although the 12- and 14-mer HDO also showed good activity. Similar to the single-stranded ASO, the HDO complex containing a 16-nucleotide ASO was significantly less active (Fig. 1c). The α-tocopherol-conjugated cRNA (Toc-cRNA) alone did not significantly decrease *ApoB* mRNA levels even at a high dose of 6 mg kg^−1^ (Supplementary Fig. 2). The direct conjugation of α-tocopherol to single-stranded ASO (Toc-ASO) (Fig. 1a) abolished the *in vivo* effect of the gapmer ASO even at a dose of 3 mg kg^−1^ (Fig. 1d).

**Figure 2:**
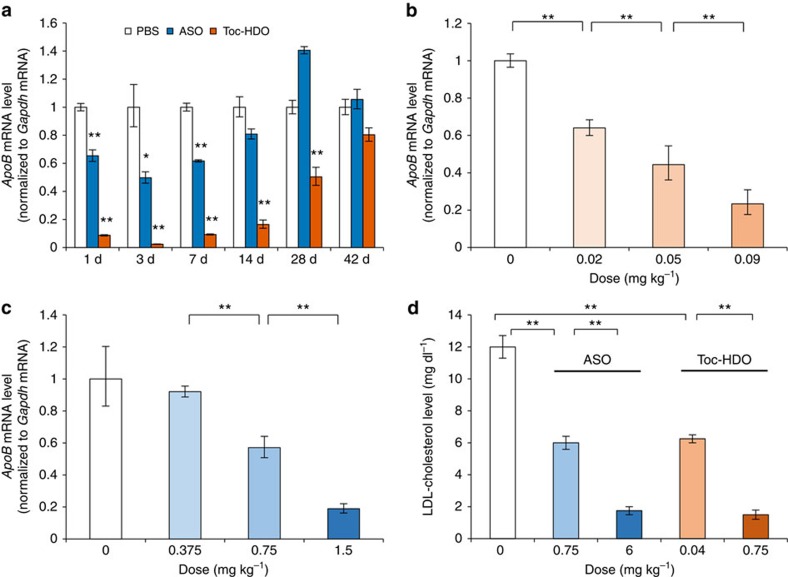
Duration of gene silencing and dose-dependent reduction by ASO and Toc-HDO-targeting *ApoB* mRNA. (**a**) qRT–PCR analyses showing *ApoB* mRNA levels in liver assayed at the indicated time points after injection of 0.75 mg kg^−1^ ASO, Toc-HDO or PBS alone. Data are expressed as mean values±s.e.m. (*n*=3, *P<0.05, **P<0.01 versus PBS). (**b**,**c**) Dose-dependent reduction of gene silencing after injection of Toc-HDO (**b**) or ASO (**c**) (*n*=3, mean values±s.e.m., **P<0.01). (**d**) Serum LDL cholesterol levels 3 d after injection of ASO, Toc-HDO or PBS alone. Data are expressed as mean values±s.e.m. (*n*=3, **P<0.01 versus PBS). Data are representative of at least three independent experiments each. *P* values were calculated from the Student's two-tailed *t*-test (**a**) or the Jonckheere–Terpstra test (**b**–**d**).

After injection, the reduction of *ApoB* mRNA in the liver was maximal on day 3, and it lasted for >1 month (Fig. 2a). The Toc-HDO (effective dose; ED_50_, 0.038 mg kg^−1^; Fig. 2b) was 22.2 times more potent than the single-stranded ASO (ED_50_, 0.841 mg kg^−1^; Fig. 2c). In addition to lowering *ApoB* mRNA, the Toc-HDO was more potent in reducing serum low-density lipoprotein (LDL) cholesterol (Fig. 2d), and the pharmacological effects lasted for >1 month for an injection dose of 0.75 mg kg^−1^ Toc-HDO (Supplementary Fig. 3).

**Figure 3:**
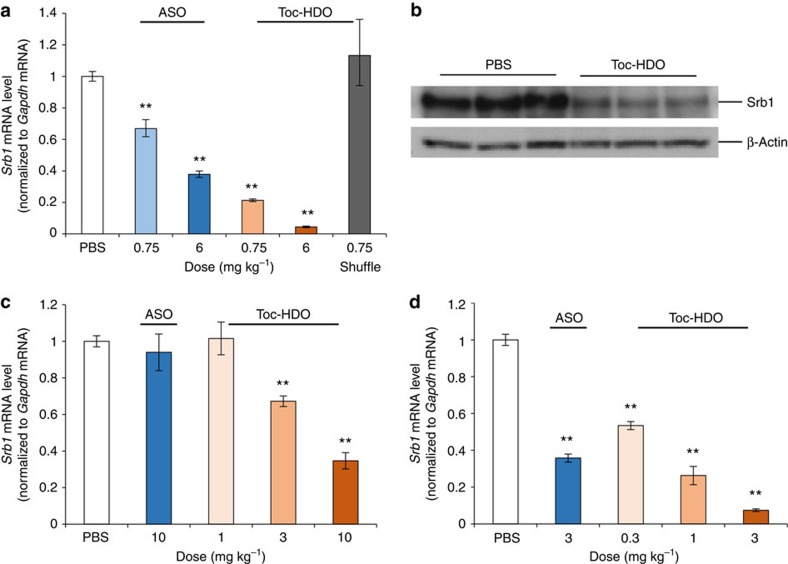
Gene-silencing effect with Toc-HDO targeting scavenger receptor class B member 1 (*Srb1*) in the liver. (**a**) qRT–PCR analysis showing *Srb1* mRNA levels in liver assayed 3 d after injection of ASO or Toc-HDO-targeting *Srb1*, shuffle sequence of Toc-HDO-targeting *Srb1* or PBS alone. Data are expressed as mean values±s.e.m. (*n*=3, **P<0.01 versus PBS). (**b**) Western blot analysis of Srb1 protein in livers from three mice 7 d after injection of 6 mg kg^−1^ Toc-HDO-targeting *Srb1* mRNA. (**c**) qRT–PCR analyses showing *Srb1* mRNA levels in liver assayed 3 d after injection of DNA/2′-MOE gapmer ASO, Toc-HDO or PBS alone. Data are expressed as mean values±s.e.m. (*n*=4, **P<0.01 versus PBS). (**d**) qRT–PCR analyses showing *Srb1* mRNA levels in liver assayed 3 d after injection of mice with DNA/cEt gapmer ASO, Toc-HDO or PBS alone. Data are expressed as mean values±s.e.m. (*n*=4, **P<0.01 versus PBS). Data are representative of at least three independent experiments each (**a**,**c**,**d**). *P* values were calculated from the Student's two-tailed *t*-test (**a**,**c**,**d**).

A significant improvement in activity was also observed when targeting another gene, scavenger receptor class B member 1 (*Srb1*) gene, in the liver (Fig. 3a,b). Furthermore, the Toc-HDO using 2′-MOE or cEt instead of LNA in the wing portion of the DNA strand also showed a similarly enhanced potency (Fig. 3c,d) for reduction of *Srb1* mRNA.

### Adverse effects of Toc-HDO

To evaluate the adverse effects of Toc-HDO, we performed repeated daily injections of the same dose (1 mg kg^−1^) of ASO or Toc-HDO for 4 consecutive days; we found a mild liver dysfunction as determined by elevated serum alanine aminotransferase (ALT) and alkaline phosphatase levels in ASO-injected mice compared with Toc-HDO-injected mice at the same doses (Supplementary Table 2). On routine histological analysis by haematoxylin–eosin staining of the liver tissues, there was no necrotic change of the hepatocytes or inflammatory changes (Supplementary Table 3). We performed another experiment in which the administered dose was adjusted to the same silencing effect at ED_50_ or ED_95_ level in ASO-injected and Toc-HDO-injected mice (Table 1). In Toc-HDO-injected mice, we found a statistically significant decrease of liver dysfunction (ALT level) observed in the parent ASO-injected mice both at ED_50_ and ED_95_ levels, probably because of the reduction of the administered dose of the nucleotide. Hepatic lipid accumulation, a known adverse effect caused by targeting *ApoB* mRNA, which was observed in Toc-HDO-injected mouse liver, was consistent with ApoB lowering (Supplementary Fig. 4). These results indicated that Toc-HDO with the same silencing effect as ASO had a less adverse effect on liver toxicity. To estimate the immune-stimulatory adverse events by administration of Toc-HDO, the levels of induction of pro-inflammatory cytokine, interferon-γ (IFN-γ) and tumour necrosis factor-α (TNF-α) were examined after injection of Toc-HDO. No increase of IFN-γ and TNF-α levels was detected in the serum (Table 1).

To address the specificity of the Toc-HDO complex, we performed microarray analysis of liver samples from mice injected with 0.75 mg kg^−1^ Toc-HDO and 6 mg kg^−1^ single-stranded ASO, doses designed to produce 95% reduction of *ApoB* mRNA. We identified 11 off-target genes with ≥84% identity to the DNA strand that were downregulated by >50% (ref. 28) in samples from Toc-HDO-injected mice (Table 2). In mice injected with the single-stranded ASO, we detected six additional genes that were downregulated by >50% (Table 2). Importantly, there was no additional off-target gene which was downregulated by >60% in Toc-HDO-injected mice relative to ASO-injected mice, suggesting that Toc-HDO did not exacerbate the effects on off-target genes compared with the parent ASO.

### Gene-silencing effect of Toc-HDO in a mouse disease model

**Figure 4:**
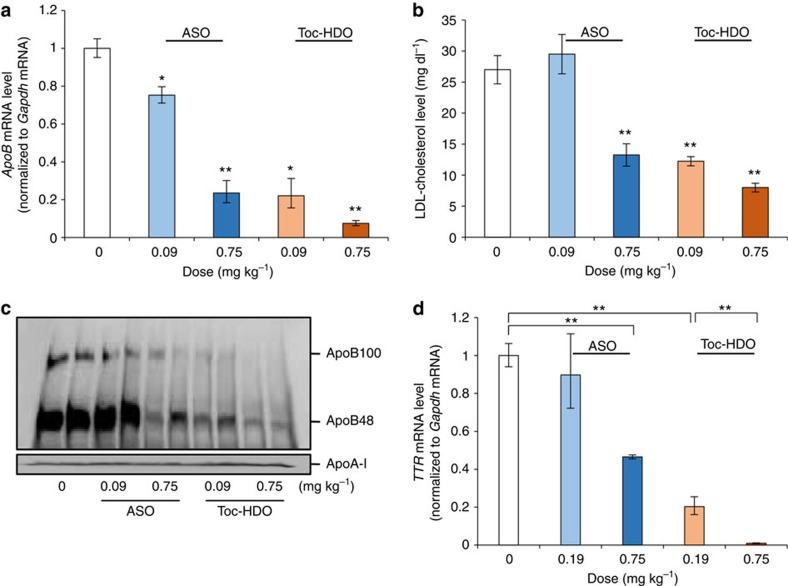
Toc-HDO mediates potent gene silencing in a mouse disease model. (**a**–**c**) Repeated injections of ASO or Toc-HDO to hypercholesterolaemic mice on a high-fat diet. qRT–PCR analyses of relative *ApoB* mRNA levels in liver (**a**), serum LDL cholesterol levels (**b**) (*n*=4, mean values±s.e.m., *P<0.05, **P<0.01 versus PBS) and western blot of serum ApoB protein (**c**). (**d**) Dose-dependent reduction of gene silencing in V30M *TTR* transgenic mice with Toc-HDO targeting *TTR* (*n*=3, mean values±s.e.m., **P<0.01). Data are representative of at least three independent experiments each (**a**,**b**,**d**). *P* values were calculated from the Student's two-tailed *t*-test (**a**,**b**) or the Jonckheere–Terpstra test (**d**).

As a mouse disease model, hypercholesterolaemic mice, produced by feeding a high-fat synthetic diet, were treated with weekly repeated doses of ASO or Toc-HDO-targeting *ApoB* mRNA (0.75 or 0.09 mg kg^−1^ per week) for 1 month. An almost 80% reduction of *ApoB* mRNA in the liver (Fig. 4a) and >50% reduction in serum LDL cholesterol levels (Fig. 4b) were achieved with both the Toc-HDO and single-stranded ASOs at the doses tested. This silencing effect was confirmed by a marked reduction in serum ApoB protein content as well (Fig. 4c). To confirm these results in another mouse disease model, we evaluated Toc-HDO in the V30M Transthyretin (*TTR*) transgenic mouse, which is the model of familial amyloid polyneuropathy. The Toc-HDO targeting the transgene *TTR* mRNA was also more potent than the parent ASO on decreasing the expression of the mutant *TTR* (Fig. 4d).

### Investigations of Toc-HDO in non-human primates

**Figure 5:**
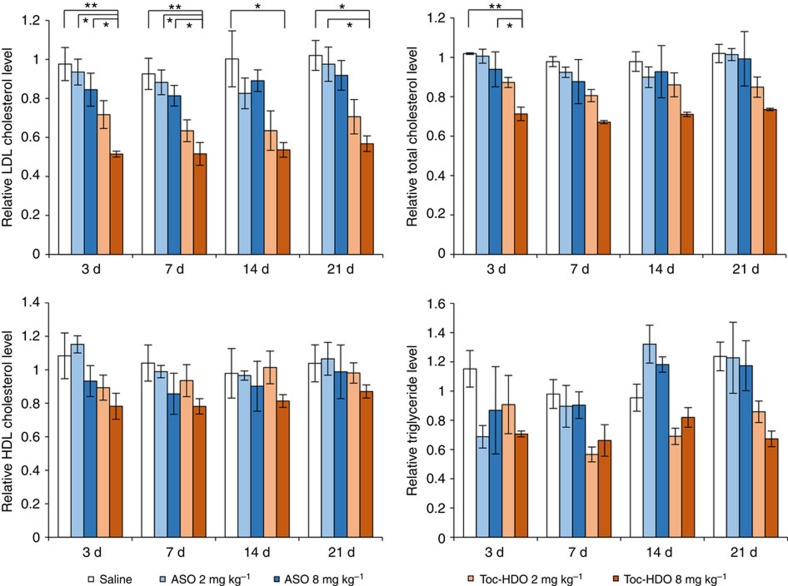
Toc-HDO mediates potent gene silencing in non-human primates. Single injections of ASO or Toc-HDO to non-human primates. Serum LDL cholesterol, total cholesterol, HDL cholesterol and triglyceride levels were shown as a ratio of pre-dose values (*n*=3, mean values±s.e.m., *P<0.05, **P<0.01.). Data are representative of two independent experiments each. *P* values were calculated from Tukey's two-tailed test.

To extend these findings to non-human primates, cynomolgus monkeys were injected with saline, 2 mg kg^−1^, or 8 mg kg^−1^ *ApoB* mRNA targeting ASO or Toc-HDO. Consistent with observations in mice, the Toc-HDO complex was more effective at reducing serum LDL cholesterol and total cholesterol compared with saline or single ASO-injected monkeys (Fig. 5, upper panels). In contrast, there was no significant change in non-ApoB-containing high-density lipoprotein (HDL) level (Fig. 5, left below panel). We observed no treatment-related adverse effects on the clinical appearance or behaviour of monkeys treated with Toc-HDO compared with saline-treated controls. Biochemical analyses of serum showed that ALT level in 8 mg kg^−1^ Toc-HDO-injected monkeys was mildly and transiently increased (Supplementary Table 4).

### Biodistribution of Toc-HDO

**Figure 6:**
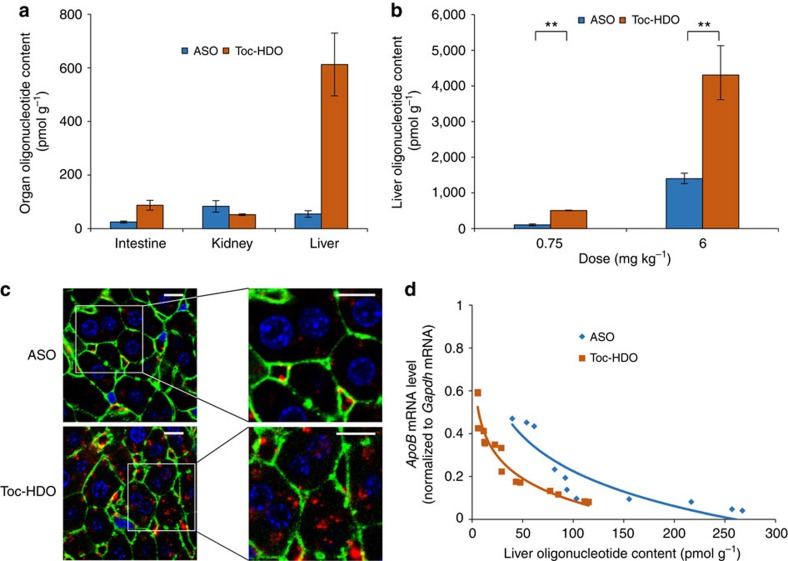
Delivery mechanisms of Toc-HDO. (**a**) Concentration of DNA/LNA gapmer in each organ 6 h after injection of 0.75 mg kg^−1^ AlexaFlour 647-labelled ASO or Toc-HDO. The other organs including brain, heart, lung, spleen and muscle had no signal. (**b**) qRT–PCR analyses of oligonucleotide levels in liver 3 d after injection (*n*=3, mean values±s.e.m., **P<0.01). (**c**) Confocal laser scanning microscopy images of mouse livers at 6 h after injection of Cy3-labelled 0.75 mg kg^−1^ ASO or Toc-HDO. Red: Cy3-labelled DNA/LNA gapmer; green: AlexaFluor 488 Phalloidin; blue: Hoechst 33342; Bar, 20 μm. (**d**) Relationship between the concentration of DNA/LNA gapmer and *ApoB* mRNA levels in the liver. The regression equation between the concentration of DNA/LNA gapmer and *ApoB* mRNA levels in liver was *y*=−0.236ln(*x*)+1.31, *R*^2^=0.815 (ASO) and *y*=−0.151ln(*x*)+0.781, *R*^2^=0.923 (Toc-HDO). Data are representative of at least three independent experiments each (**a**,**b**,**d**). *P* values were calculated from Student's two-tailed *t*-test (**b**).

We investigated the mechanism underlying the enhanced silencing ability of Toc-HDO. A relatively selective accumulation of fluorescence-labelled Toc-HDO in the liver was detected by histological analysis (Supplementary Fig. 5) and by quantification of the signal intensities (Fig. 6a). The absolute tissue concentration of the DNA strand in the liver of mice injected with 0.75 mg kg^−1^ Toc-HDO was ∼5.0 times that observed for ASO by quantitative real-time PCR assay (Fig. 6b) and enzyme-linked immunosorbent assay (ELISA) (Supplementary Fig. 6). Increased accumulation of Toc-HDO compared with the parent ASO was also confirmed by histological analysis with fluorescence-labelled Toc-HDO. The fluorescence signal in the cytosol of hepatocytes and non-parenchymal cells was more robust in Toc-HDO-injected mouse liver (Fig. 6c). Because the increase in the silencing effect of Toc-HDO relative to ASO (22.2-fold) at ED_50_ was more than the increase in oligonucleotide delivery to the liver (5.0-fold), we analysed the correlation between delivered oligonucleotide content and level of target mRNA in the liver of mice (Fig. 6d). The differences between the fitted curves showed that the silencing efficacy of Toc-HDO delivered to the liver (effective concentration; EC_50_, 6.4 pmol g^−1^) was 4.8 times higher than that of ASO (EC_50_, 30.9 pmol g^−1^), which could be a result of enhanced delivery to hepatocytes over non-parenchymal cells^29^, or of increased silencing effect of Toc-HDO after the uptake of hepatocytes.

### Binding molecules of Toc-HDO in serum

**Figure 7:**
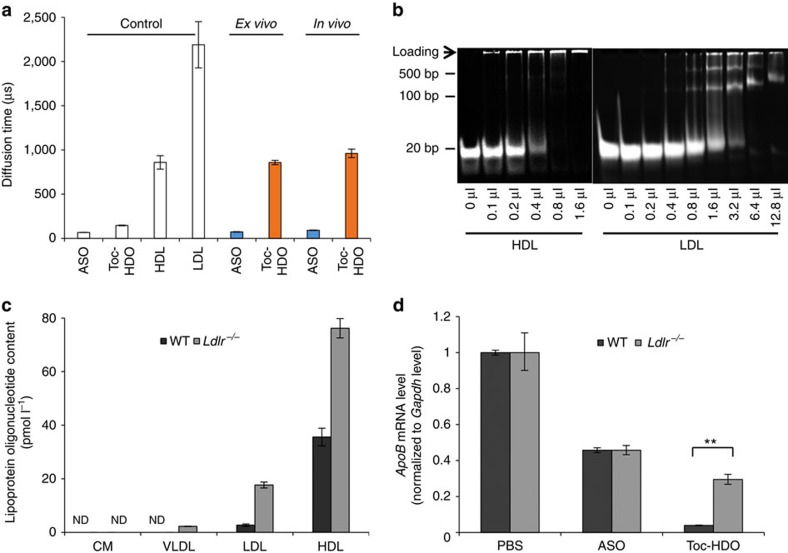
Incorporation of Toc-HDO into lipoproteins. (**a**) Diffusion time of Cy3-labelled oligonucleotides in HDL fraction of serum (*ex vivo*) or the serum extracted from mice 10 min after injection of 3 mg kg^−1^ Cy3-labelled ASO or Toc-HDO (*in vivo*) determined by fluorescence correlation spectroscopic analysis (*n*=10, mean values±s.e.m.). HDL or LDL fraction (control) was stained using Nile Red. (**b**) Gel-shift assay of Toc-HDO with extracted mouse HDL or LDL. (**c**) Oligonucleotide concentration of serum lipoprotein fractions 30 min after injection of 6 mg kg^−1^ AlexaFlour 647-labelled Toc-HDO (*n*=3, mean values±s.e.m.). ND, not detected. (**d**) qRT–PCR analyses of *ApoB* mRNA levels in liver 3 d after injection of 0.75 mg kg^−1^ Toc-HDO into wild-type or *Ldlr*^−/−^ mice. Data are expressed as mean values±s.e.m. (*n*=3, **P<0.01). Data are representative of at least three independent experiments each (**a**,**c**,**d**). *P* values were calculated from Student's two-tailed *t*-test (**d**).

We explored whether this hepatic tropism was related to interactions with a carrier molecule in serum. We investigated the carrier molecule of Toc-HDO to the liver using a mixture of Toc-HDO and collected mouse lipoprotein fractions from serum (*ex vivo*) and serum from Toc-HDO-injected mouse (*in vivo*). The results of fluorescence correlation spectroscopy indicated that the estimated size of the serum molecule that bound to Toc-HDO (both *ex vivo* and *in vivo*) was close to that of HDL, which is the major component of mouse serum lipoproteins. In contrast, the size of ASO was much smaller and was similar to that of albumin (Fig. 7a). Next, we examined the binding of Toc-HDO and lipid profiles using high-performance gel-shift assays. The endogenous HDL fraction was isolated by ultracentrifugation from the sera of wild-type mice, whereas the endogenous LDL fraction was collected from the sera of LDL receptor (LDLR)-deficient (*Ldlr*)^−/−^ mice, because wild-type mice only had small amounts of LDL in their sera. The gel-shift assays demonstrated the binding of Toc-HDO to both separated LDL and HDL in a saturable manner (Fig. 7b). Furthermore, we found that injected Toc-HDO was present with some of the serum proteins and lipids including HDL, and there was no free Toc-HDO in mouse serum (Supplementary Fig. 7). We also investigated the binding of Toc-HDO to LDL and HDL fractions, which were collected by fast protein liquid chromatography (FPLC) from the fluorescent-labelled Toc-HDO-injected wild-type or *Ldlr*^−*/*−^ mouse serum (Fig. 7c). Toc-HDO distributed both LDL and HDL fractions with predominance to the HDL fraction.

To examine the uptake mechanism by hepatocytes, we injected Toc-HDO into *Ldlr*^−*/*−^ mice. The suppression of target *ApoB* mRNA was decreased in *Ldlr*^−*/*−^ mice compared with wild-type mice (Fig. 7d). This indicated that uptake of Toc-HDO by hepatocytes was mediated, at least in part, through LDLR. Together, these data show that conjugation with α-tocopherol results in the binding of Toc-HDO to serum lipoproteins and a more specific and efficient delivery to the liver along with physiological pathway of vitamin E^30^.

### Processing mechanisms of Toc-HDO

**Figure 8:**
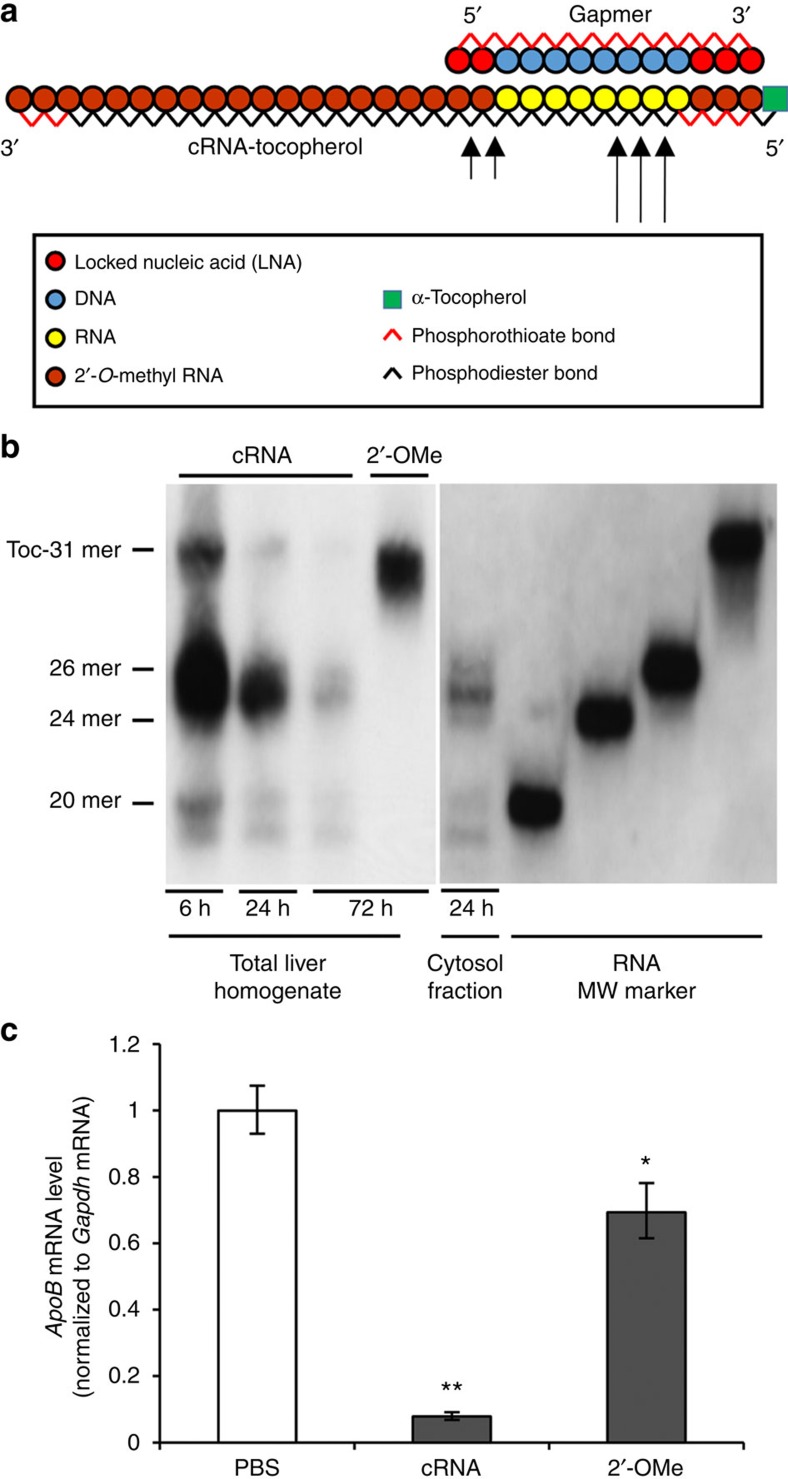
Intracellular processing mechanisms of Toc-HDO. (**a**) Schematic illustration of the construction of 13-mer DNA/31-mer cRNA Toc-HDO. Arrows: presumed cleavage sites of the 31-mer cRNA. (**b**) Northern blot analysis of 31-mer cRNA sequences of Toc-HDO in livers from 6 mg kg^−1^ of Toc-HDO or RNase-resistant Toc-HDO (that is, Toc-HDO cRNA molecules in which all of the RNA was replaced by 2′-*O*-methyl RNA (2′-OMe cRNA))-injected mice. cRNA, Toc-HDO with unmodified cRNA; 2′-OMe, Toc-HDO with 2′-OMe cRNA. (**c**) qRT–PCR analyses of *ApoB* mRNA levels in liver injected with 0.75 mg kg^−1^ Toc-HDO, Toc-HDO with 2′-OMe cRNA or PBS alone. cRNA, Toc-HDO with unmodified cRNA; 2′-OMe, Toc-HDO with 2′-OMe cRNA (*n*=3, mean values±s.e.m., *P<0.05, **P<0.01 versus PBS). Data are representative of at least three independent experiments each (**c**). *P* values were calculated from Student's two-tailed *t*-test (**c**).

Next, we investigated how the processing of Toc-HDO relates to the silencing mechanism. To analyse processing of the RNA strand, 13-mer DNA/31-mer cRNA with an 18-mer 2′-*O*-methyl RNA overhang on the 3′ end was used for the detection of cRNA in northern blot analysis (Fig. 8a). Northern blot analysis of liver from mice treated with Toc-HDO with 13-mer DNA/31-mer cRNA (similar potency as 13-mer DNA/13-mer cRNA, Supplementary Fig. 8) revealed that the full-length cRNA was almost non-detectable by 24 h after injection. The detected cRNA fragments indicated that the cRNA was cleaved by two different cleavage sites at 2/3/4 base pairs and 9/10 base pairs from the 5′ end of the unmodified RNA of the heteroduplex (Fig. 8a,b). This suggested that the cleaved cRNA fragments were too short to remain bound to the DNA strand, resulting in unwinding from the DNA strand. It should be noted that the major cleavage sites (2/3/4 base pairs from the 5′ end of unmodified RNA) are different from that reported for recombinant human RNase H1 (refs 31, 32, 33, 34). When all RNA of cRNA molecules were replaced by RNase-resistant 2′-*O*-methyl RNA (Fig. 8b), the *in vivo* silencing effect of Toc-HDO was markedly reduced (Fig. 8c), indicating that cleavage of the cRNA is important for efficient silencing with Toc-HDO.

## Discussion

ASO with the RNase H mechanism of action is typically composed of 13–25–nucleotide-long single-stranded DNA, whereas siRNA is composed of 21- to 30-nucleotide-long double-stranded RNA. Previous studies have shown that conjugation of fatty acids and cholesterol to siRNAs and micro-RNA targeting single-stranded ASOs enhances the half-life in circulation, cellular uptake and activity of the antisense agent^25^,^35^,^36^,^37^. To extend these studies, we tried to directly conjugate α-tocopherol to single-stranded ASO-targeting *ApoB* mRNA, but the silencing activity of the ASO was much reduced in mouse liver compared with the unconjugated ASO (Fig. 1d). This was likely a result of the conjugated α-tocopherol interfering with the access of the ASO to the target mRNA in the nucleus by tethering the ASO to lipid membranes or other molecules within cells^27^. Lipid-conjugated siRNA, which was designed with a cleavable linker or nucleotide to release the lipid from oligonucleotides in the cells, had more potency^25^,^38^,^39^. In our previously reported case, α-tocopherol conjugated to the 5′ terminus of the guide strand of 27/29-mer siRNA was processed by Dicer to generate 21/21-mer mature siRNA, resulting in a release of α-tocopherol moiety from the oligonucleotide in the cytoplasm^25^. Surprisingly, we found that the hybridizing α-tocopherol-conjugated cRNA to DNA/LNA gapmer markedly enhanced the activity of the parent ASO (Fig. 1b). We found that the cRNA was cleaved in the liver by specific nucleases (Fig. 8b). The major cleavage sites were different from that reported for recombinant human RNase H1 and may reflect cleavage by another endonuclease such as RNase T2-like RNase in lysosome^40^. Inhibition of the cleavage of the cRNA interfered with silencing ability (Fig. 8c), indicating that release of cRNA with conjugated lipid from oligonucleotide in the cells is a key processing step for the parent ASO activity^41^.

Recently, several approaches such as liposomes or LNPs have been reported to deliver siRNA^8^,^42^,^43^,^44^ and ASO^45^ to the liver, and they showed strong silencing effect of target gene *in vivo*. However, LNP-formulated siRNAs still have limitations in the clinic^9^. LNP-formulated siRNAs are more efficient at escaping endosomal compartments, but they are prone to immune activation^9^ and their immunogenicity and cytotoxicity has been reported^46^,^47^. LNPs are more complex to manufacture^9^ and the large particle size of LNPs (40–70 nm)^8^,^44^,^47^ restricts their biodistribution to tissues with open vasculatures including liver and tumour^9^. In comparison, the preparation of Toc-HDO is significantly less complex, and they were not found to be immunostimulatory in our evaluations (Table 1).

We previously conjugated α-tocopherol to siRNA (Toc-siRNA) targeting mouse *Apo*B mRNA and obtained increased delivery of siRNA in mouse liver^25^. Its ED_50_ was ∼2 mg kg^−1^, and the knockdown effect of the target gene returned to the baseline level 4 days after injection^25^. In contrast, the ED_50_ of Toc-HDO was 0.038 mg kg^−1^, and the reduction of *ApoB* mRNA in the liver and the decrease of serum LDL cholesterol lasted for >1 month. Although direct comparison of Toc-siRNA and Toc-HDO is difficult because of differences in the target sequence and chemistry of the oligonucleotides, Toc-HDOs are more efficient for *in vivo* applications than Toc-siRNAs.

In cynomolgus monkeys, larger amounts of Toc-HDO were found to be necessary to achieve a similar reduction of serum LDL cholesterol than in mice (Figs 2d and 5). In this study, we used the same sequence of the DNA strand of Toc-HDO-targeting mouse *ApoB* mRNA as reported previously^23^. The parent ASO binds cynomolgus monkey and mouse *ApoB* mRNA with 100% homology. In this previous study, 32 mg kg^−1^ of a single dose of this ASO was required to reduce serum LDL cholesterol level by 70% in monkeys, whereas the same ASO reduced non-HDL cholesterol by 70% in mice at a dose of 2.5 mg kg^−1^ (ref. 23). Broad experience with phosphorothioate gapmer ASO in pre-clinical animal models has shown that they generally exhibit lower potency in monkeys as compared with mice and humans. Yu *et al.*^48^ reported cross-species comparison of *in vivo* pharmacokinetics/pharmacodynamics relationships of Mipomersen, a phosphorothioate DNA/MOE gapmer ASO. The EC_50_ of this ASO in liver tissue was 101±32 μg g^−1^ in mice, 119±15 μg g^−1^ in transgenic mice containing the human *APOB* transgene and 81±122 μg g^−1^ in humans, whereas EC_50_ was 300±191 μg g^−1^ in cynomolgus monkeys^48^. These results suggested that ASOs are less active in monkeys as compared with mice and humans. The origins of these effects are not obvious but could be a result of differences in binding to individual plasma and cell surface proteins in different species resulting in differential intracellular trafficking of ASOs^49^,^50^.

Liver safety is an important concern for clinical applications of all antisense therapeutic modalities^51^. Sequence-dependent and hybridization-dependent off-target effects have been observed, especially with shorter ASOs. The microarray gene expression analysis showed several downregulated genes predicted to be because of off-target hybridization of HDO (Table 2). In addition, sequence-dependent and hybridization-independent immune-stimulatory adverse effects mediated by activation of toll-like innate receptors^52^,^53^ can also be a concern. Finally, a sequence- and hybridization-independent chemical property of nucleotide analogues and chemical modifications can cause liver dysfunction^51^,^54^,^55^,^56^,^57^. Although our data suggested that Toc-HDO did not have additional off-target genes relative to the parent ASO, and exhibited no immune-stimulatory adverse effects, there was a minor but significant liver dysfunction probably caused by LNA and/or α-tocopherol conjugation (Supplementary Table 2). However, as it is dose dependent, this chemical adverse effect was decreased compared with the parent ASO at the dose producing the same silencing effect (Table 1).

In summary, the Toc-HDO molecular technique can be applied to any ASOs previously reported, and it can improve the silencing effect of the ASOs by ∼20-fold when targeting the liver. This improved efficacy and no increase in adverse effects observed with Toc-HDO helps justify the use of the second strand despite the slightly enhanced complexity associated with clinical development. Furthermore, conjugation of another appropriate drug delivery molecule to HDO can extend this technology to target other organs or cells. Our ligand-conjugated DNA/RNA heteroduplex opens up a new horizon for human gene therapy as a novel class of oligonucleotide drugs.

## Methods

### Design and synthesis of Toc-HDO

A series of DNA/LNA, DNA/2′-MOE or DNA/cEt gapmer and cRNAs of different lengths (12- to 31-mers) were designed to target mouse *ApoB* mRNA (NM_009693), which was the same as human *APOB* mRNA (NM_000384)^23^, mouse organic anion transporter 3 (*Oat3*) mRNA (NM_031194) or mouse *Srb1* mRNA (NM_016741)^22^.

A series of gapmers were synthesized by Gene Design (Osaka, Japan) and ISIS Pharmaceuticals (Carlsbad, CA). The sequences of the gapmer targeting *ApoB* mRNA were as follows: 12-mer gapmer, 5′-G*C*a*t*t*g*g*t*a*t*T*C-3′; 13-mer gapmer, 5′-G*C*a*t*t*g*g*t*a*t*T*C*A-3′; 14-mer gapmer, 5′-A*G*C*a*t*t*g*g*t*a*t*T*C*A-3′; and 16-mer gapmer, 5′-C*A*G*c*a*t*t*g*g*t*a*t*t*C*A*G-3′, where lowercase italic letters represent DNA, uppercase bold letters represent LNA (capital C denotes LNA methylcytosine) and asterisks represent phosphorothioate linkages. The sequence of another 14-mer gapmer targeting mouse *ApoB* mRNA^58^ was 5′-C*C*A*a*c*c*a*a*t*t*t*C*T*C-3′. The sequence of the non-*ApoB*-related 13-mer gapmer targeting mouse *Oat3* mRNA was 5′-G*A*a*g*g*t*c*a*t*g*G*C*A-3′, and that of the 13-mer gapmer targeting the human *TTR* mRNA was 5′-C*G*t*a*g*t*t*g*t*a*A*T*C-3′.

The sequences of the gapmer targeting *Srb1* mRNA were as follows: 13-mer gapmer with LNA, 5′-C*A*g*t*c*a*t*g*a*c*T*T*C-3′; 14-mer gapmer with cEt, 5′-T*C*a*g*t*c*a*t*g*a*c*t*T*C-3′; and 20-mer gapmer with 2′-MOE, 5′-G*C*T*T*C*a*g*t*c*a*t*g*a*c*t*T*C*C*T*T-3′, where asterisks represent phosphorothioate linkages, lowercase italic letters represent DNA and uppercase bold letters represent LNA (capital C denotes LNA methylcytosine) in the 13-mer gapmer, cEt in the 14-mer gapmer and 2′-MOE in the 20-mer gapmer.

The shuffle sequence of the 13-mer gapmer targeting *ApoB* mRNA is 5′-T*G*t*c*t*c*t*g*c*c*T*G*G-3′, and that of the 13-mer gapmer targeting *Srb1* mRNA is 5′-A*C*c*g*a*t*a*c*t*g*C*T*T-3′.

A series of cRNAs were synthesized by Hokkaido System Science (Sapporo, Japan). The sequences of the cRNAs targeting *ApoB* mRNA are as follows: 12-mer cRNA, 5′-g*a*AUACCAAU*g*c-3′; 13-mer cRNA, 5′-u*g*a*AUACCAAU*g*c-3′; 13-mer 2′-OMe cRNA, 5′-u*g*a*auaccaau*g*c-3′; 14-mer cRNA, 5′-u*g*a*AUACCAAU*g*c*u-3′; 16-mer cRNA, 5′-c*u*g*AAUACCAAUG*c*u*g-3′; 31-mer cRNA, 5′-u*g*a*AUACCAAUgcuacgcauacgcacca*c*c*a-3′; and 31-mer 2′-OMe cRNA, 5′-u*g*a*auaccaaugcuacgcauacgcacca*c*c*a-3′, where uppercase letters represent RNA, lowercase letters represent 2′-*O*-methyl sugar modification and asterisks represent phosphorothioate linkages. The sequence of another 14-mer cRNA targeting mouse *ApoB* mRNA was 5′-g*a*g*AAAUUGGU*u*g*g-3′. The sequence of the 13-mer non-*ApoB*-related cRNA targeting mouse *Oat3* mRNA is 5′-u*g*c*CAUGACCU*u*c-3′, and the sequence of the 13-mer cRNA complementary to the gapmer targeting the human *TTR* mRNA is 5′-c*c*a*GGCAGAGA*c*a-3′. The sequences of the cRNAs targeting *Srb1* mRNA are as follows: 13-mer cRNA, 5′-g*a*a*GUCAUGAC*u*g-3′; 14-mer cRNA, 5′-g*a*AGUCAUGACU*g*a-3′; and 20-mer cRNA, 5′-a*a*g*g*a*AGUCAUGACU*g*a*a*g*c-3′. The shuffle sequence of the 13-mer cRNA targeting *ApoB* mRNA is 5′-g*a*u*UACAACUA*c*g-3′, and that of the 13-mer gapmer targeting *Srb1* mRNA is 5′-a*a*g*CAGUAUCG*g*u-3′.

Cy3 or AlexaFluor 647 fluorophores were covalently bound to the 5′ ends of DNA/LNA gapmers, and α-tocopherol was covalently bound to the 5′ ends of DNA/LNA gapmers or cRNAs. For the generation of HDO, equimolar amounts of DNA and RNA strands were heated in PBS (Sigma-Aldrich, St Louis, MO) at 95 °C for 5 min and slowly cooled to room temperature.

### Mouse studies

Wild-type Crlj:CD1 (ICR) mice or C57BL/6 mice aged 4–5 weeks (Oriental Yeast, Tokyo, Japan), 5-week-old B6.129S7-*Ldlr* (tm1Her)/J (*Ldlr*^−*/*−^) mice (Jackson Laboratory, ME) and human mutant *TTR* transgenic mice (expressing the Val30Met TTR protein) were kept on a 12-h light/dark cycle in a pathogen-free animal facility with free access to food and water. ASO or Toc-HDO was administered to the mice by tail-vein injection on the basis of body weight. All oligonucleotides were formulated in PBS, which was also used as the control. The oligonucleotides were administered to mice by a single injection or repeated injections. Animal experiments were performed at the Tokyo Medical and Dental University, except for animal experiments with 2′-MOE and cEt gapmer, which were performed at ISIS Pharmaceuticals. All experiments were conducted with more than three mice, and these procedures were in accordance with the ethical and safety guidelines for animal experiments of Tokyo Medical and Dental University (#0140144A) and American Association for the Accreditation of Laboratory Animal Care. The mouse model of hypercholesterolaemia was produced by feeding mice a high-fat synthetic diet (CE-2 plus 20% w/w beef tallow powder and 1.25% cholesterol, 10 kGy irradiation, CLEA Japan, Tokyo, Japan) from 3 weeks before injection. The 0.02–6 mg kg^−1^ oligonucleotides were administered to wild-type mice by a single injection. The 0.09 or 0.75 mg kg^−1^ oligonucleotides were administered to hypercholesterolaemia model mice, and the injections were repeated once a week for 4 weeks. For hypercholesterolaemia model mice, sera were collected 7 days (d) after the final injection for measurement of LDL cholesterol levels and western blot analysis. For postmortem analyses, mice were deeply anesthetized first with intraperitoneally administered 60 mg kg^−1^ pentobarbital, and then killed by transcardiac perfusion with PBS after confirming the absence of blink reflex.

### Non-human primate studies

Cynomolgus monkeys were housed in individual cages at the Tsukuba Primate Medical Center, National Institute of Biomedical Innovation. The animals (three males per dose level) received 10-min intravenous infusion of either 2 or 8 mg kg^−1^ ASO, Toc-HDO or saline. Blood samples were collected for haematology and blood chemistry analyses from the femoral veins before the administration and at days 3, 7, 14 and 21 after administration. All experiments were conducted with more than three Cynomolgus monkeys, and these procedures were in accordance with the ethical and safety guidelines for animal experiments at the Tokyo Medical and Dental University (#0150097A).

### Quantitative real-time PCR assay

Total RNA was extracted from mouse liver by using Isogen (Nippon Gene, Tokyo, Japan). To detect mRNA, DNase-treated RNA (2 μg) was reverse transcribed with SuperScript III and Random Hexamers (Life Technologies, Carlsbad, CA). To detect short oligonucleotides, including DNA/LNA gapmer, quantitative real-time (qRT)–PCR analysis was performed using the TaqMan MicroRNA Reverse Transcription Kit (Applied Biosystems, Foster City, CA) and a Light Cycler 480 Real-Time PCR Instrument (Roche Diagnostics, Mannheim, Germany). The primers and probes for DNA/LNA gapmers, mouse *ApoB*, *Srb1*, glyceraldehyde-3-phosphate dehydrogenase (*Gapdh*; NM_008084), transthyretin (*Ttr*; NM_013697), superoxide dismutase 1 (*Sod1*; NM_011434), sno-234 (*Snord70*; NR_028554) and human *TTR* (NM_000371) genes were designed by Applied Biosystems.

### Western blot analysis

Mouse serum (4 μl) was diluted with 12 μl of PBS, and mouse liver (50 mg) was homogenated using 125 μl of homogenate buffer (20 mM Tris-HCl (pH 7.4), 0.1% SDS, 0.1% Triton X-100, 0.01% sodium deoxycholate and 1 × Complete protease inhibitor cocktail (Roche Diagnostics)). Samples (16 μl) were mixed with 4 μl of Laemmli sample buffer (Bio-Rad, Hercules, CA) and then denatured at 95 °C for 2 min. Total proteins were separated by electrophoresis on a 5–20% gradient polyacrylamide gel (ATTO Corporation, Tokyo, Japan) and transferred onto polyvinylidene difluoride membranes. Blots were probed with goat primary antibodies against Apolipoprotein A-I (1:500, sc-23605, Santa Cruz Biotechnology, Santa Cruz, CA) and Apolipoprotein B (1:500, sc-11795, Santa Cruz Biotechnology), or Scavenging receptor SRB1 (1:2,000, ab52629, Abcam, Cambridge, UK) and β-actin (1:500, ab6276, Abcam), and then incubated with anti-goat secondary antibody (1:2,000, sc-2020, Santa Cruz Biotechnology), anti-rabbit secondary antibody (1:2,000, sc-2004, Santa Cruz Biotechnology) or anti-mouse secondary antibody (1:2,000, sc-2005, Santa Cruz Biotechnology) conjugated with horseradish peroxidase. Blots were visualized with SuperSignal West Femto Maximum Sensitivity Substrate (Thermo Fisher Scientific, Waltham, MA) and analysed by a ChemiDoc System (Bio-Rad).

### Isolation of the lipoprotein fraction from serum

The lipoprotein fraction was prepared by ultracentrifugation according to the following method^59^. First, a half volume of a solution of density 1.182 g ml^−1^ was layered onto one volume of mouse serum and centrifuged for 3.6 h at 337,000*g* at 16 °C. The half volume of the upper solution was set aside for use in experiments as the LDL fraction. The one volume of the lower solution was mixed with a half volume of a solution of density 1.478 g ml^−1^ and centrifuged for 7.5 h at 266,000*g* at 16 °C. The half volume of the upper solution obtained after this second centrifugation contained HDL and was used in experiments as the HDL fraction.

### Fast protein liquid chromatography (FPLC)

Lipoprotein profiles of isolated mouse lipoprotein fraction were analysed by an online dual enzymatic method for simultaneous quantification by FPLC at Skylight Biotech (Akita, Japan)^60^. Mouse serum (10 μl) 30 min after injection of AlexaFluor 647-conjugated 6 mg kg Toc-HDO was diluted with saline up to a volume of 200 μl and loaded onto FPLC columns (TSKgel LipopropakXL, Tosoh Co., Ltd, Tokyo, Japan), followed by simultaneous and continuous detection of total cholesterol. The flow-through was subfractionated into 20 tubes for 60 s each, with 262.5 μl in each tube. Samples were taken from each subfraction: tubes 1 and 2 (over 75.0 nm) contained the chylomicron-sized fraction from serum; tubes 3–7 (64.0–31.3 nm) contained the very-LDL-sized fraction; tubes 8–13 (28.6–16.7 nm) contained the LDL-sized fraction; and tubes 14–20 (under 15.0 nm) contained the HDL-sized fraction. Each sample was used to measure the concentration of DNA/LNA gapmer as described below.

### DNA/LNA gapmer concentration in serum or each organ

Mice were injected with AlexaFluor 647-conjugated ASO or Toc-HDO, and 6 h later various organs (brain, heart, lung, liver, kidney, spleen, intestine and muscle) and serum were harvested. The signal intensity of AlexaFluor 647 in the serum or homogenate of each organ was measured by i-control (Tecan, Männedorf, Switzerland), and then the concentration of DNA/LNA gapmer was calculated.

### Gel-shift assay

For *ex vivo* samples, Toc-HDO (100 pmol) was added to 0–66 μl of mouse serum, 0–1.6 μl of HDL fraction from mouse serum or 0–12.8 μl of LDL fraction from *Ldlr*^−*/*−^ mouse serum. For *in vivo* samples, serum was collected from mouse 5 min after injection of 0.75 mg kg^−1^ Toc-HDO. The samples were resolved by electrophoresis on a 15% polyacrylamide gel for 60 min at 100 V. The oligonucleotides were visualized under ultraviolet after staining the gel with ethidium bromide in Tris–borate–EDTA buffer.

### Fluorescence correlation spectroscopy analysis

To elucidate the interacting carrier molecule in the serum, we first incubated Cy3-labelled ASO or Toc-HDO (100 pmol) for 20 min at 37 °C with mouse HDL fraction (99 μl) (‘*ex vivo*' samples). Next, we intravenously injected 3 mg kg^−1^ Cy3-labelled ASO or Toc-HDO to the mouse, and corrected serum 10 min after injection (‘*in vivo*' samples). The measurements were performed using the ConfoCor 3 module in combination with the LSM 510 (Carl Zeiss MicroImaging, Göttingen, Germany) equipped with the C-Apochromat × 40/1.2 W objective. A HeNe laser (543 nm) was used for Cy3-labelled ASO and Toc-HDO excitation in PBS or serum, and excitation of the Nile Red (Tokyo Chemical Industry, Tokyo, Japan)-labelled HDL or LDL fraction. Emission was filtered through a 560- to 615-nm band-pass filter. Samples were placed into an 8-well Lab-Tek chambered slide (Nalgene Nunc International, Rochester, NY), and diffusion time was measured at room temperature. Autocorrelation curves obtained from 10 measurements with a sampling time of 20 s were fitted with the ConfoCor 3 software package to determine the diffusion time of samples.

### Northern blot analysis

Total RNA was extracted from mouse liver using Isogen II (Nippon Gene). Total RNA (30 μg) was separated by electrophoresis in an 18% polyacrylamide urea gel and transferred to a Hybond-N^+^ membrane (Amersham Biosciences, Piscataway, NJ). The blot was hybridized with a probe corresponding to the cRNA sequence, or with the mouse U6 micro-RNA sequence (internal control), which had been labelled with digoxigenin-ddUTP using a DIG Oligonucleotide 3′-End Labelling Kit, 2nd Generation (Roche Diagnostics). The sequence of the DNA probe for detecting cRNA was 5′-TGGTGCGTATGCGTAGCATTGGTATTCA-3′. The signals were visualized using the Gene Images CDP-star Detection Kit (Amersham Biosciences).

### Isolation of cytosolic fraction from liver tissue

Subcellular fractionation of hepatocytes was conducted by centrifugation by the following method^61^. Liver samples (50 mg each) were homogenized in 1 ml of buffer containing 0.32 M sucrose, 1 mM dithiothreitol, 20 mM 4-(2-hydroxyethyl)-1-piperazineethanesulfonic acid (HEPES) homogenization buffer (pH 7.2) and 1 mM phenylmethylsulfonyl fluoride, by 10 strokes of a tight-fitting glass homogenizer. The homogenate was centrifuged at 1,200*g* for 15 min, and the supernatant was used as the cytosolic fraction.

### Melting temperature measurement

Ultraviolet melting experiments were performed using a Shimadzu UV-1650PC spectrometer (Shimadzu, Kyoto, Japan) equipped with a melting temperature (*T*_m_) analysis accessory. Equimolecular amounts of two single-stranded oligonucleotides were dissolved in 10 mM sodium phosphate buffer (pH 7.2) containing 100 mM NaCl to give a final strand concentration of 4 μM. The oligonucleotides were annealed at 90 °C and slowly cooled to room temperature. The melting profile was recorded at 260 nm in the forward and reverse direction from 5 to 90 °C at a scan rate of 0.5 °C min^−1^.

### Evaluation of adverse effects

A single dose, 0.75 or 6 mg kg^−1^ ASO or 0.04 or 0.75 mg kg^−1^ Toc-HDO, was injected into the tail veins of mice. Sera were collected 3 d after injection, and blood chemistry was assessed. For the repeated-dose study, 1 mg kg^−1^ ASO or Toc-HDO in PBS was injected into the tail vein of ICR mice for 4 consecutive days. Blood samples were collected 24 h after the final injection, haematology and blood chemistry were assessed and organs were collected for the pathological study described below.

### Histopathological analyses

For pathological analyses, organs were postfixed in 4% paraformaldehyde (Wako Pure Chemical Industries, Osaka, Japan) in PBS for 6 h, embedded in paraffin, cut into 4-μm-thick sections using a Leica CM 3050S cryostat (Leica Microsystems, Wetzlar, Germany) and stained with haematoxylin and eosin (Muto Pure Chemicals, Tokyo, Japan). To analyse liver lipid accumulation, mice were injected with 0.75 mg kg^−1^ ASO or 0.04 mg kg^−1^ Toc-HDO and 7 d later the liver was removed and fixed in 4% paraformaldehyde in PBS for 12 h and snap-frozen in liquid nitrogen. The liver samples were sectioned (10 μm) and stained with filtered Oil Red O (Muto Pure Chemicals) at 37 °C for 10 min. Nuclei were counterstained with Mayer haematoxylin solution for 10 min. The slides were analysed using an Olympus AX80 Automatic Research Photomicroscope (Olympus, Tokyo, Japan).

To analyse the biodistribution of Toc-HDO, 0.75 mg kg^−1^ Cy3-labelled ASO or Toc-HDO in PBS was injected into the mouse tail veins. Tissues were fixed in 4% paraformaldehyde in PBS for 12 h and snap-frozen in liquid nitrogen. Tissue sections (10 μm) were prepared with a Leica CM3050 S cryostat (Leica Microsystems). The sections were stained with Hoechst 33342 (Sigma-Aldrich) to visualize nuclei and with 13 nmol l^−1^ AlexaFluor 488 phalloidin (Life Technologies) to visualize cell membrane, and then they were analysed using a LSM 510 confocal microscope (Carl Zeiss MicroImaging).

### Enzyme-linked immunosorbent assay

ELISA-based detection of ASOs was performed by the following method^62^. Frozen liver tissues of Toc-HDO-treated mice were mechanically homogenized for 2 min at 30 oscillations s^−1^. Total protein concentrations of the resultant solutions were adjusted to 4 mg ml^−1^. To prepare six standard solutions for the calibration curve, we hybridized known concentrations of ASOs to cRNAs by denaturing them at 95 °C for 5 min and overnight annealing at 37 °C. Then, an oligonucleotide-untreated liver homogenate was added to serially diluted duplex-containing solutions to yield final concentrations of oligonucleotides ranging from 25.6 pM to 200 nM. These standard samples were incubated with a nuclease solution containing ribonuclease H (Takara Bio, Otsu, Japan) and exonuclease T (New England Biolabs, Ipswich, MA) at 25 °C for 3 h and heated at 70 °C for 40 min. Equal amounts of 23-mer biotinylated template DNA solution and each standard or sample solution were mixed and incubated at 37 °C for 1 h on Reacti-Bind NeutrAvidin-coated 96-well plates. The plates were then washed three times with washing buffer, the 9-mer probe DNA was added and the plates were incubated at 15 °C for 3 h. Subsequently, 200 μl of anti-digoxigenin-AP (1:2,000) was added, and the plates were incubated at 37 °C for 1 h. After washing, AttPhos Substrate (Promega, Fitchburg, WI) solution was added. After 15 min, the fluorescence intensity was determined using a SpectraMax M2e microplate reader (Molecular Devices, Sunnyvale, CA). The linear range of the ELISA system was 25.6 pM to 200 nM with *R*^2^>0.987.

The levels of IFN-γ and TNF-α in the blood samples were analysed using the Mouse IFN-γ ELISA Kit (R&D Systems, Minneapolis, MN) and Mouse TNF-α ELISA Kit (R&D Systems) according to the manufacturers' protocol.

### Microarray experiments

Mouse livers were harvested 3 d after injection of 6 mg kg^−1^ ASO or 0.75 mg kg^−1^ Toc-HDO. Total RNA was extracted from the livers using the mirVana miRNA Isolation Kit (Life Technologies) according to the manufacturers' protocol to separate procedures for large RNA isolation. Microarray analyses (50 ng of total RNA) were performed using SurePrint G3 Mouse GE 8 × 60 K (Agilent Technologies, Santa Clara, CA).

### Statistical analysis

All data represent mean±s.e.m. Student's two-tailed *t*-tests were used to determine the significance of differences between two groups in qRT–PCR assays and analyses of lipoprotein levels in serum. Tukey's two-tailed tests were used to determine the significance of differences to multiple comparisons in qRT–PCR, haematology and blood chemistry analyses. The Jonckheere–Terpstra test was used to determine the significance of differences in dose-dependent reduction analyses of *ApoB* or *TTR* mRNA levels. Analysis of covariance was used to determine the significance of differences between fitted curves of concentration of DNA/LNA gapmer and liver *ApoB* mRNA levels in liver. The regression equation of the dose–response relationship was *y*=−0.528ln(*x*)+0.4083, *R*^2^=0.999 (ASO) and *y*=−0.293ln(*x*)−0.4588, *R*^2^=0.999 (Toc-HDO).

## Additional information

**How to cite this article:** Nishina, K. *et al.* DNA/RNA heteroduplex oligonucleotide for highly efficient gene silencing. *Nat. Commun.* 6:7969 doi: 10.1038/ncomms8969 (2015).

## Supplementary Material

Supplementary Information Supplementary Figures 1-8 and Supplementary Tables 1-4

## Figures and Tables

**Table 1:**
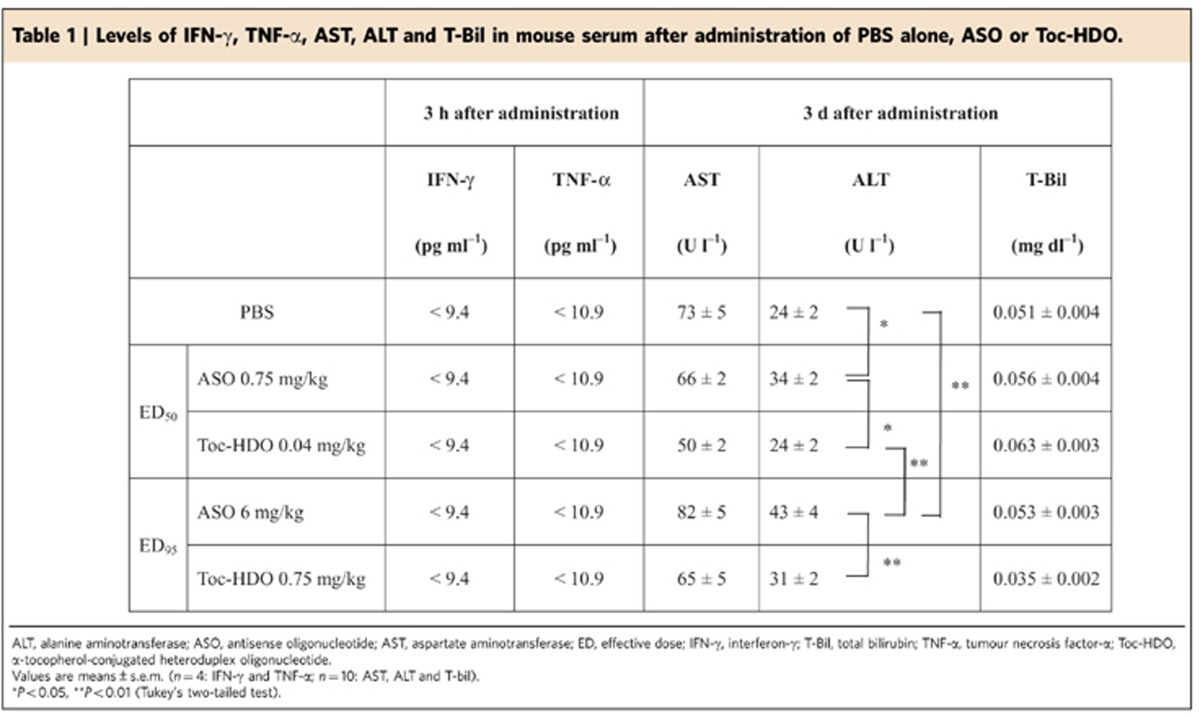
Levels of IFN-γ, TNF-α, AST, ALT and T-Bil in mouse serum after administration of PBS alone, ASO or Toc-HDO.

**Table 2:**
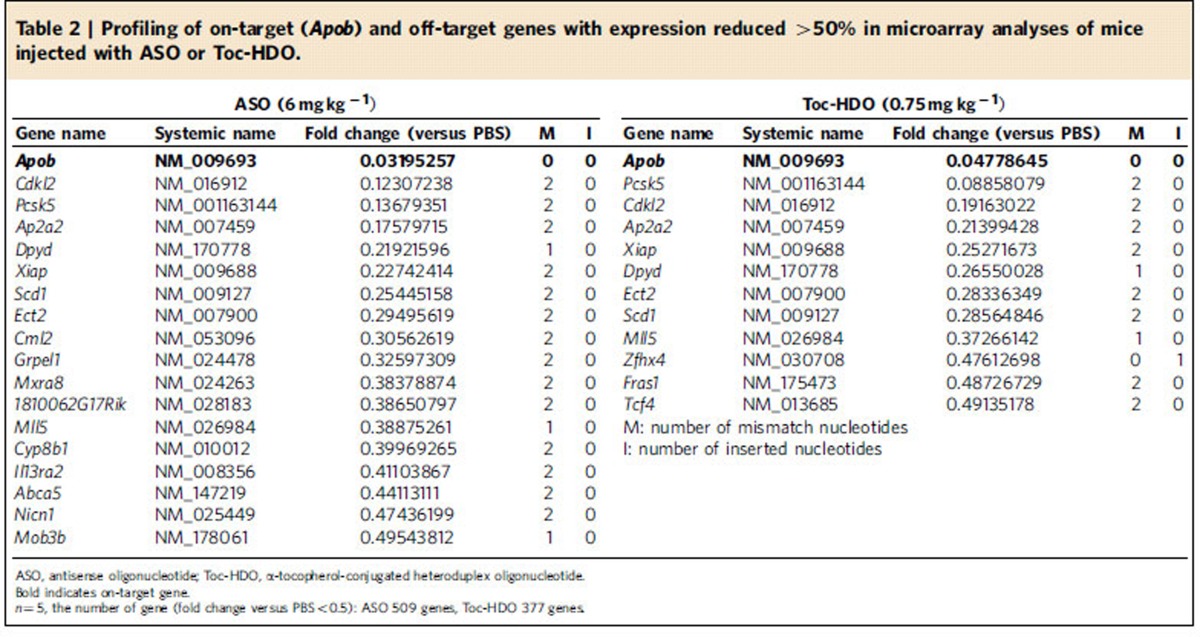
Profiling of on-target (*Apob*) and off-target genes with expression reduced >50% in microarray analyses of mice injected with ASO or Toc-HDO.
