# Supplementary material for: DNA/RNA heteroduplex oligonucleotide for highly efficient gene silencing
Source: Nat Commun. 2015 Aug 10;6:7969. doi: 10.1038/ncomms8969 (PMC4918363; doi:10.1038/ncomms8969)
Supplement: Supplementary Information — Supplementary Figures 1-8 and Supplementary Tables 1-4 [file ncomms8969-s1.pdf]

Supplementary Figures

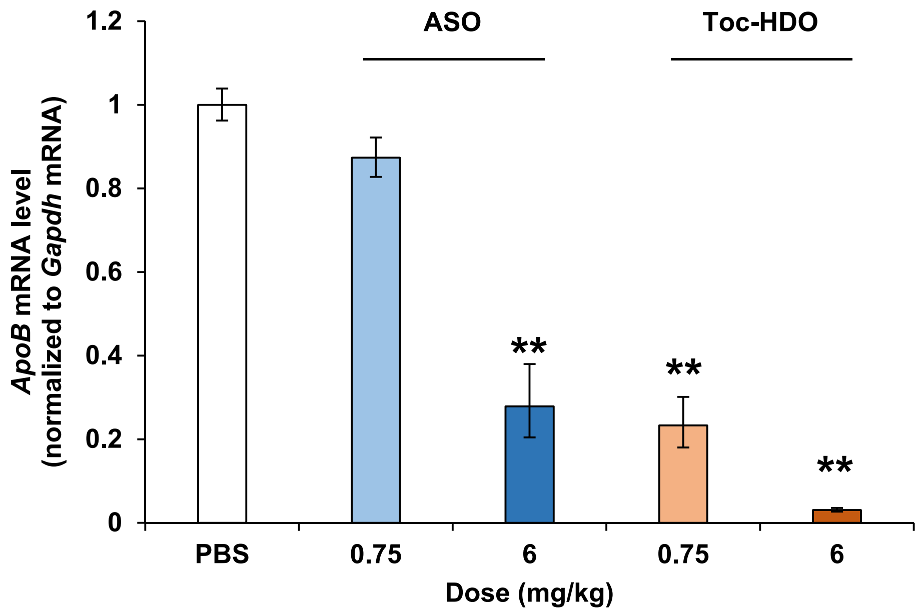

Supplementary Figure 1. The efficacy of another Toc-HDO targeting *ApoB* mRNA.

qRT-PCR analyses of *ApoB* mRNA levels in liver 3 d after injection of 0.75 and 6 mg/kg another sequence of Toc-HDO targeting *ApoB* mRNA. Data are expressed as mean values ± s.e.m. ($n = 3$, ** $P < 0.01$ versus PBS). Data are representative of at least three independent experiments each. *P* values were calculated from Student's two-tailed *t*-test.

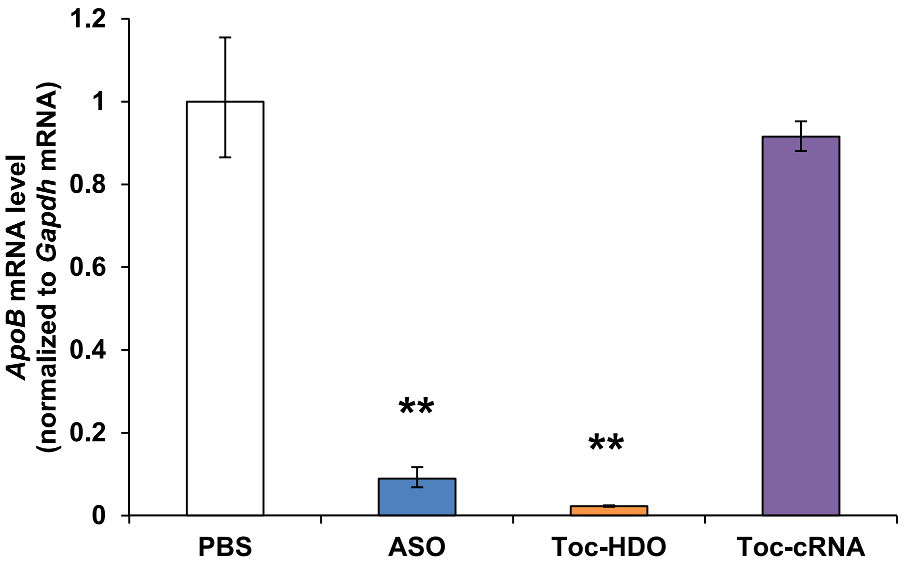

Supplementary Figure 2. α-tocopherol–conjugated complementary RNA does not have a silencing effect.

qRT-PCR analyses showing *ApoB* mRNA levels in liver assayed 3 d after injection of 6 mg/kg ASO, α-tocopherol–conjugated DNA/RNA heteroduplex oligonucleotide (Toc-HDO), α-tocopherol–conjugated cRNA (Toc-cRNA), or PBS alone. Data are expressed as mean values ± s.e.m. (n = 3, ** $P < 0.01$ versus PBS). Data are representative of at least three independent experiments each. P values were calculated from Student's two-tailed t-test.

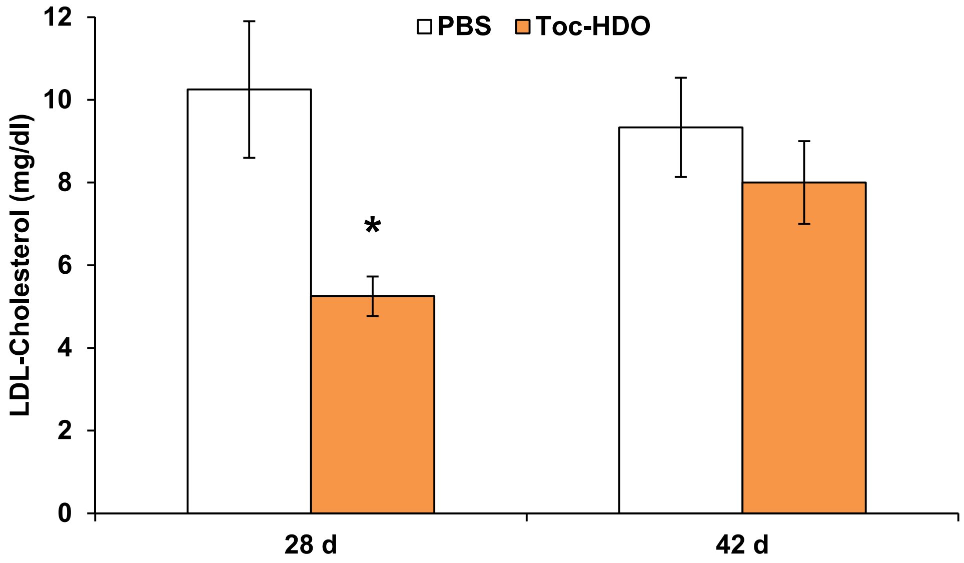

Supplementary Figure 3. Duration of serum LDL–cholesterol reduction after *ApoB* mRNA silencing by Toc-HDO targeting *ApoB* gene.

LDL–cholesterol levels in serum from 0.75 mg/kg Toc-HDO injected mice at indicated time points. Data are expressed as mean values ± s.e.m. (n = 4 at 28 d, 3 at 42 d, * $P <$ 0.05 versus PBS). Data are representative of at least three independent experiments each. P values were calculated from Student's two-tailed t-test.

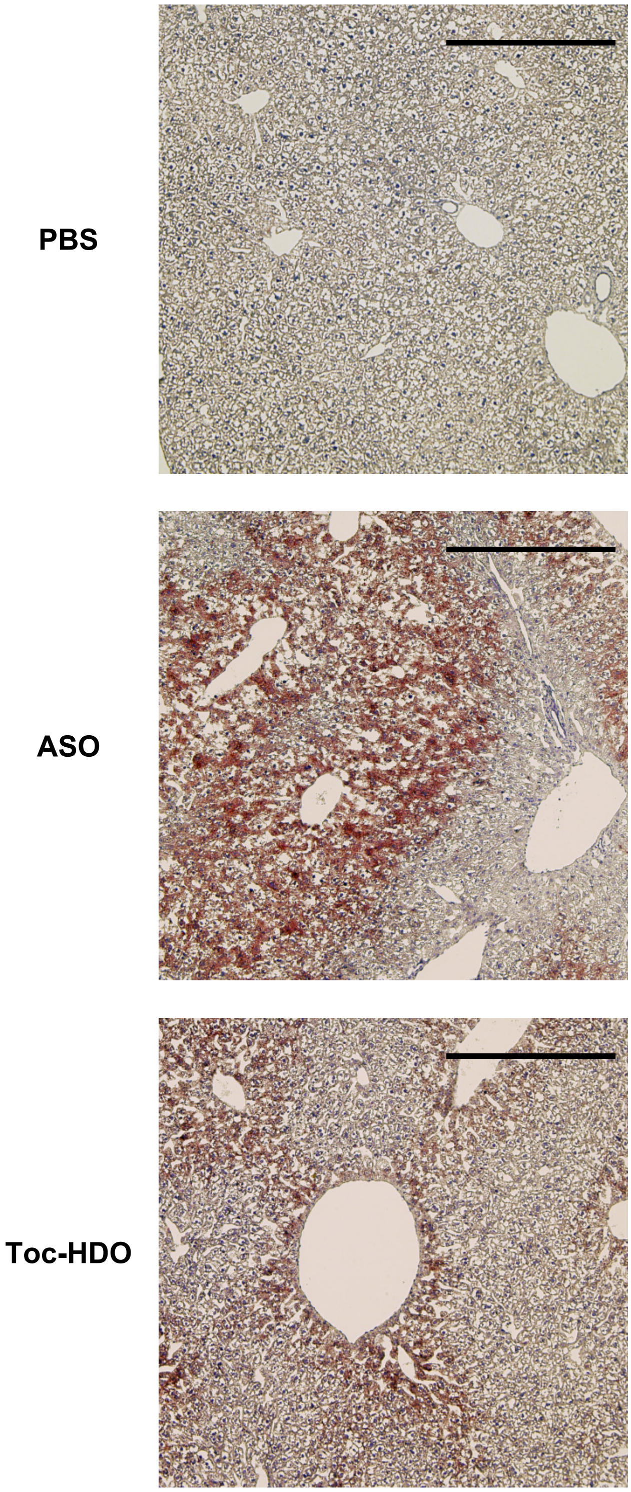

Supplementary Figure 4. Histological analysis for accumulation of hepatic lipid droplet.

The hepatic steatosis observed in 0.04 mg/kg Toc-HDO injected mice (*ApoB* reduction rate; 0.39) was not more than that of 0.75 mg/kg ASO injected mice (*ApoB* reduction rate; 0.41). Oil Red O staining of liver 3 d after injection of 0.75 mg/kg ASO, 0.04 mg/kg Toc-HDO, or PBS alone. Bar = 50 µm.

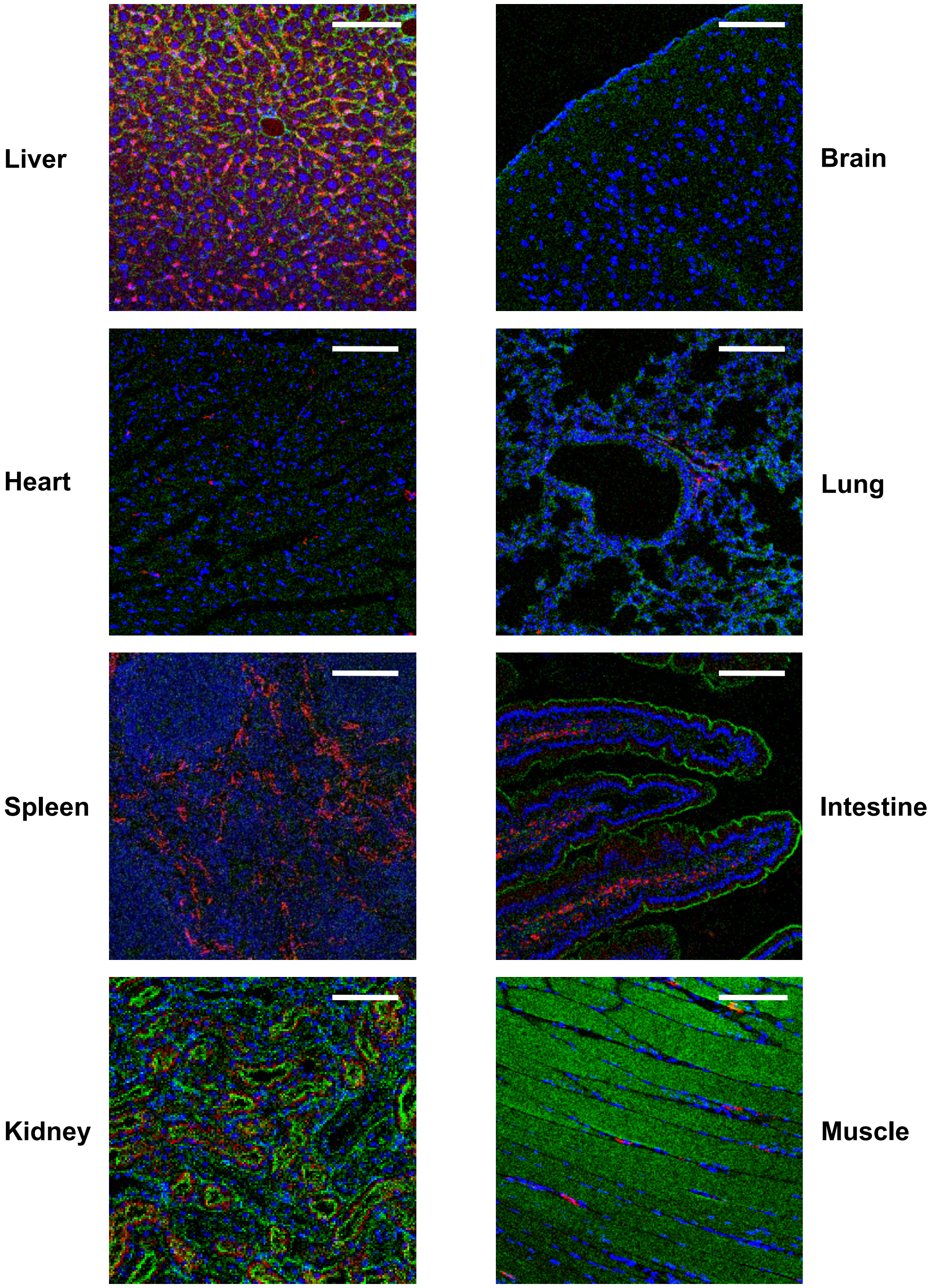

Supplementary Figure 5. Biodistribution of Toc-HDO.

Fluorescence micrographs of various mice organs 6 h after injection of 0.75 mg/kg Cy3-labeled Toc-HDO. Red: Cy3-labeled DNA/LNA gapmer; Green: AlexaFluor 488 Phalloidin; Blue: Hoechst 33342; Bar = 100 µm.

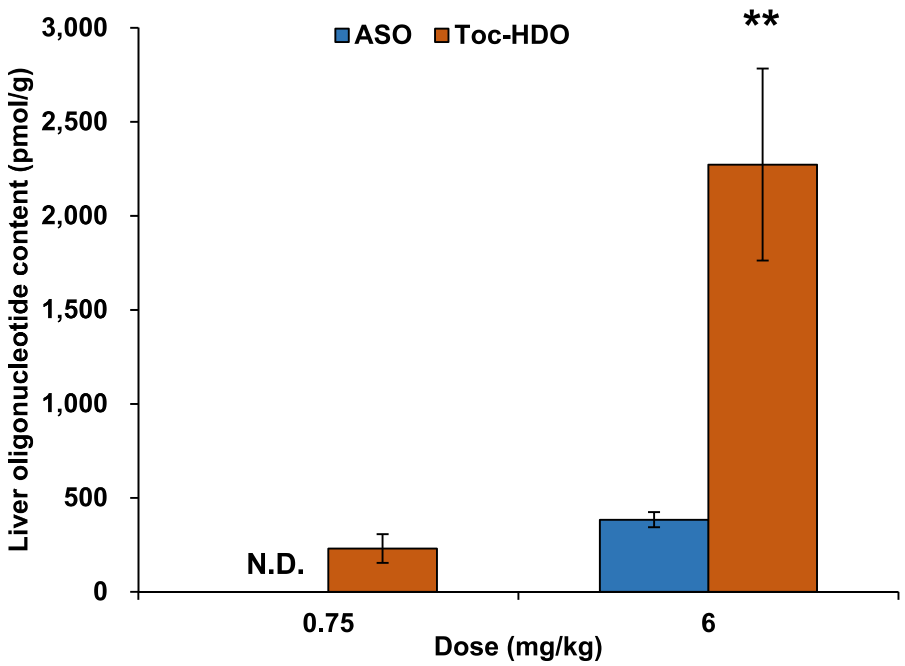

Supplementary Figure 6. ELISA analyses of DNA/LNA gapmer in liver.

The content of DNA/LNA gapmer in liver of mice after injection of 0.75 or 6 mg/kg of ASO or Toc-HDO was measured by enzyme-linked immunosorbent assay (ELISA). Data are expressed as mean values ± s.e.m (n = 3, ** $P < 0.01$ versus ASO). Data are representative of at least three independent experiments each. P values were calculated from Student's two-tailed t-test. N.D., not detected.

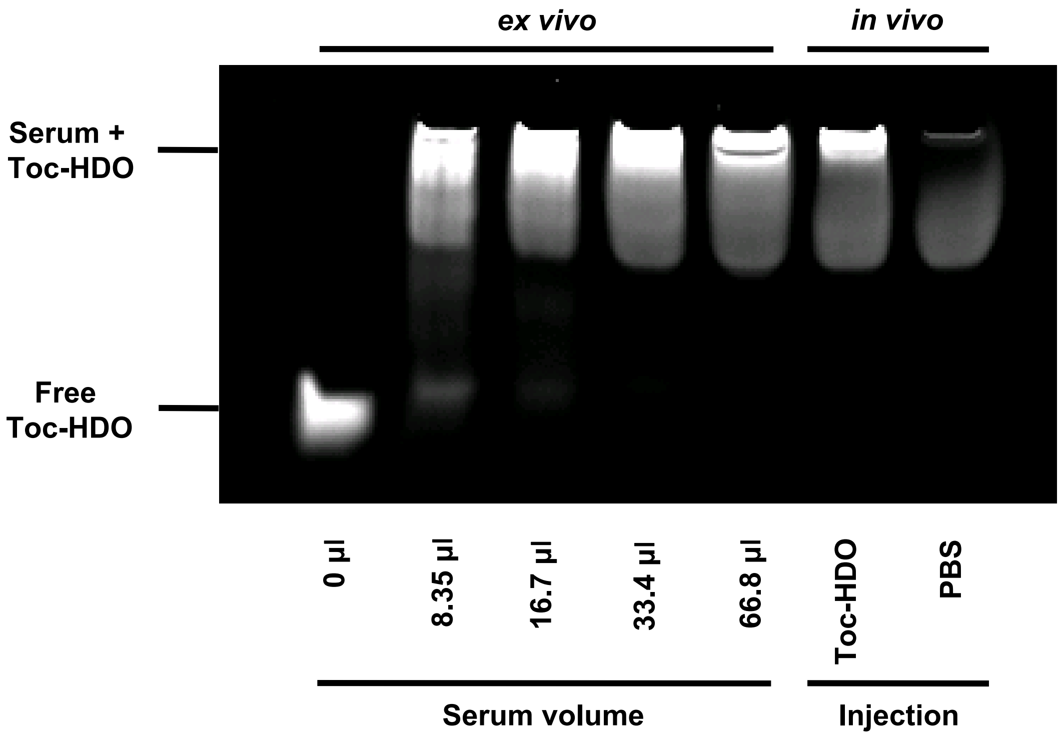

Supplementary Figure 7. Gel-shift assay of Toc-HDO with mouse serum.

For *ex vivo* samples, Toc-HDO (100 pmol) was incubated with different volumes of the mouse serum. For *in vivo* samples, serum was collected from mouse 5 min after injection of 0.75 mg/kg Toc-HDO or PBS only. The electrophoretic mobility of free Toc-HDO was examined by using a 15% polyacrylamide gel.

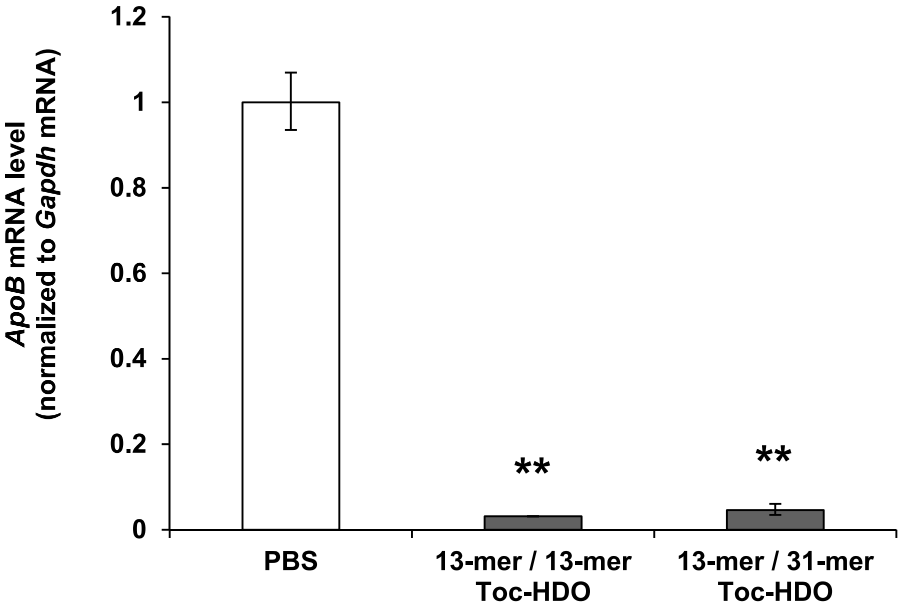

Supplementary Figure 8. The efficacy of Toc-HDO which had long Toc-cRNA.

qRT-PCR analyses of *ApoB* mRNA levels in liver 3 d after injection of 0.75 mg/kg Toc-HDO. Toc-HDO of 13-mer DNA/31-mer cRNA was similarly potent as that of 13-mer DNA/13-mer cRNA. Data are expressed as mean values ± s.e.m. (n = 3, ** $P < 0.01$ versus PBS). Data are representative of at least three independent experiments each. P values were calculated from Student's two-tailed t-test.

Supplementary Tables

Supplementary Table 1. The melting temperature (T_m) of HDO and Toc-HDO targeting mouse *Apolipoprotein B* (*ApoB*) mRNA.

T_m (°C)	12-mer	13-mer	14-mer
HDO	47	57	61
Toc-HDO	47	54	59
Toc-HDO with 2′-OMe cRNA		53	

Supplementary Table 2. Hematology and blood chemistry analyses of mice after repeated intravenous injection of 1 mg/kg ASO, Toc-HDO, or PBS alone for 4 consecutive days.

	PBS	ASO	Toc-HDO
Hematology			
Red blood cells ($\times 10^6$/μl)	9.57 ± 0.27	9.69 ± 0.30	9.82 ± 0.23
Hemoglobin (g/dl)	14.2 ± 0.7	14.5 ± 0.4	14.7 ± 0.2
Hematocrit (%)	52.7 ± 3.0	54.1 ± 1.2	55.5 ± 0.6
White blood cells ($\times 10^3$/μl)	6.55 ± 0.71	5.91 ± 0.40	6.15 ± 0.30
Platelets ($\times 10^3$/μl)	1235 ± 80	1151 ± 65	1196 ± 68
Blood Chemistry			
Aspartate aminotransferase (IU/l)	62 ± 8	56 ± 7	82 ± 12
Alanine aminotransferase (IU/l)	26 ± 2	30 ± 3	53 ± 5$^{**, \#\#}$
Total bilirubin (mg/dl)	0.040 ± 0.003	0.050 ± 0.009	0.041 ± 0.005
Alkaline phosphatase (IU/l)	812 ± 11	832 ± 21	1335 ± 44$^{**, \#\#}$
Lactase dehydrogenase (IU/l)	318 ± 30	321 ± 26	360 ± 31
Cholinesterase (IU/l)	23 ± 1	25 ± 1	25 ± 1
Urea nitrogen (mg/dl)	36.0 ± 1.2	35.1 ± 1.5	32.7 ± 1.1
Creatinine (mg/dl)	0.129 ± 0.001	0.140 ± 0.010	0.130 ± 0.010
Creatine kinase (IU/l)	261 ± 84	227 ± 65	308 ± 99
Glucose (mg/dl)	175 ± 10	171 ± 4	174 ± 4
Total protein (g/dl)	4.79 ± 0.08	4.90 ± 0.10	4.70 ± 0.10
Albumin (g/dl)	3.35 ± 0.04	3.44 ± 0.06	3.31 ± 0.06

Values represent mean ± s.e.m. (n = 9). $^{\#\#}$ $P < 0.01$ versus ASO, ** $P < 0.01$ versus PBS (Tukey's test).

Supplementary Table 3. Histological findings in mice after repeated intravenous injection of 1 mg/kg ASO or Toc-HDO for 4 consecutive days.

		PBS			ASO			Toc-HDO		
Organ	Findings	+1	+2	+3	+1	+2	+3	+1	+2	+3
heart	necrosis (myocardium)	0	0	0	0	0	0	0	0	0
	hemorrhage (myocardium)	0	0	0	0	0	0	0	0	0
liver	necrosis (hepatocytes)	0	0	0	0	0	0	0	0	0
kidney	cell death (proximal renal tubular epithelium)	0	0	0	0	0	0	0	0	0
adrenal gland	cell death (cortex)	0	0	0	0	0	0	0	0	0
spleen	increased neutrophils (red pulp)	0	0	0	0	0	0	0	0	0
	hypertrophy (histiocytes, marginal zone)	0	0	0	0	0	0	0	0	0
	cell death (red pulp)	0	0	0	0	0	0	0	0	0
mesenteric lymph node	hypertrophy (histiocytes, marginal zone)	0	0	0	0	0	0	0	0	0
bone marrow, femur	increased megakaryocytes	0	0	0	0	0	0	0	0	0
testis	cell infiltration (neutrophils, interstitium)	0	0	0	0	0	0	0	0	0
stomach	cell infiltration (neutrophils, submucosa)	0	0	0	0	0	0	0	0	0

$n = 4$, +1: slight; +2: moderate; +3: marked.

Supplementary Table 4. Serum aspartate aminotransferase (AST), alanine aminotransferase (ALT), urea nitrogen (UN) and creatinine (Cre) levels of non-human primates after single intravenous injection of 2 or 8 mg/kg ASO, Toc-HDO, or saline alone.

		Saline	ASO		Toc-HDO	
			2 mg/kg	8 mg/kg	2 mg/kg	8 mg/kg
AST (IU/l)	Pre	45 ± 6	31 ± 4	41 ± 6	36 ± 4	46 ± 4
	day 3	28 ± 6	38 ± 8	33 ± 4	38 ± 11	65 ± 13
	day 7	35 ± 3	38 ± 4	39 ± 5	46 ± 7	54 ± 7
	day 14	53 ± 6	46 ± 2*	48 ± 8	31 ± 6	72 ± 8*
	day 21	36 ± 4	43 ± 7	40 ± 4	41 ± 4	57 ± 7
ALT (IU/l)	Pre	38 ± 14	35 ± 7	43 ± 16	25 ± 7	57 ± 18
	day 3	34 ± 15	37 ± 7	36 ± 12	25 ± 5	75 ± 11
	day 7	32 ± 12	35 ± 6	42 ± 15	34 ± 3	69 ± 14
	day 14	28 ± 6	35 ± 6	39 ± 12	26 ± 1	82 ± 27
	day 21	25 ± 7	33 ± 9	40 ± 12	25 ± 4	94 ± 33
UN (mg/dl)	Pre	18.0 ± 4.2	15.8 ± 2.3	18.6 ± 2.5	14.0 ± 1.0	13.3 ± 0.8
	day 3	17.9 ± 0.9	17.7 ± 3.6	17.9 ± 1.7	16.5 ± 1.0	17.2 ± 2.8
	day 7	16.1 ± 1.5	17.8 ± 1.0	17.4 ± 2.1	15.1 ± 1.6	12.7 ± 0.6
	day 14	15.6 ± 0.5	16.4 ± 1.5	17.7 ± 0.5	15.0 ± 1.1	14.5 ± 1.8
	day 21	13.1 ± 1.1	16.6 ± 1.3	16.4 ± 2.5	14.6 ± 1.1	14.7 ± 1.2
Cre (mg/dl)	Pre	0.60 ± 0.03	0.60 ± 0.00	0.50 ± 0.03	0.60 ± 0.03	0.60 ± 0.06
	day 3	0.60 ± 0.10	0.60 ± 0.07	0.50 ± 0.00	0.50 ± 0.03	0.60 ± 0.07
	day 7	0.60 ± 0.03	0.60 ± 0.03	0.50 ± 0.06	0.60 ± 0.10	0.60 ± 0.06
	day 14	0.50 ± 0.03	0.60 ± 0.00	0.50 ± 0.07	0.50 ± 0.03	0.60 ± 0.03
	day 21	0.60 ± 0.00	0.60 ± 0.03	0.50 ± 0.00	0.50 ± 0.03	0.60 ± 0.03

Values represent mean ± s.e.m. (n = 3). * $P < 0.05$ versus Pre (Student's t-test).
